# Socio-ecological implications of modifying rotation lengths in forestry

**DOI:** 10.1007/s13280-015-0747-4

**Published:** 2016-01-07

**Authors:** Jean-Michel Roberge, Hjalmar Laudon, Christer Björkman, Thomas Ranius, Camilla Sandström, Adam Felton, Anna Sténs, Annika Nordin, Anders Granström, Fredrik Widemo, Johan Bergh, Johan Sonesson, Jan Stenlid, Tomas Lundmark

**Affiliations:** Department of Wildlife, Fish and Environmental Studies, Swedish University of Agricultural Sciences (SLU), 901 83 Umeå, Sweden; Department of Forest Ecology and Management, Swedish University of Agricultural Sciences (SLU), 901 83 Umeå, Sweden; Department of Ecology, Swedish University of Agricultural Sciences (SLU), Box 7044, 750 07 Uppsala, Sweden; Department of Political Science, Umeå University, 901 87 Umeå, Sweden; Southern Swedish Forest Research Centre, Swedish University of Agricultural Sciences (SLU), Rörsjövägen 1, Box 49, 230 53 Alnarp, Sweden; Department of Historical, Philosophical and Religious Studies, Umeå University, 901 87 Umeå, Sweden; Department of Forest Genetics and Plant Physiology, Umeå Plant Science Centre, Swedish University of Agricultural Sciences (SLU), 901 83 Umeå, Sweden; Department of Forestry and Wood Technology, Linnaeus University, 351 95 Växjö, Sweden; Skogforsk, Uppsala Science Park, 751 83 Uppsala, Sweden; Department of Forest Mycology and Plant Pathology, Swedish University of Agricultural Sciences (SLU), Box 7026, 750 07 Uppsala, Sweden

**Keywords:** Climate change, Forest damage, Non-timber forest products, Production, Recreation, Timber

## Abstract

The rotation length is a key component of even-aged forest management systems. Using Fennoscandian forestry as a case, we review the socio-ecological implications of modifying rotation lengths relative to current practice by evaluating effects on a range of ecosystem services and on biodiversity conservation. The effects of shortening rotations on provisioning services are expected to be mostly negative to neutral (e.g. production of wood, bilberries, reindeer forage), while those of extending rotations would be more varied. Shortening rotations may help limit damage by some of today’s major damaging agents (e.g. root rot, cambium-feeding insects), but may also increase other damage types (e.g. regeneration pests) and impede climate mitigation. Supporting (water, soil nutrients) and cultural (aesthetics, cultural heritage) ecosystem services would generally be affected negatively by shortened rotations and positively by extended rotations, as would most biodiversity indicators. Several effect modifiers, such as changes to thinning regimes, could alter these patterns.

## Introduction

A major proportion of the world’s managed forests are subjected to even-aged management systems where stands are harvested through clearcutting. The stands are subsequently regenerated naturally or artificially, and allowed to grow (with or without thinning) until final felling. A key parameter describing even-aged forest management systems is rotation length, i.e. the time elapsed between two final fellings. The choice of a rotation length is an integral part of any forest management regime (Curtis 1997). It is dictated by the goals of forest management and biophysical factors. In many even-aged systems, the main management goal is the production of wood-based commodities such as saw timber, pulpwood or woody biomass. In this context, the optimal rotation is mostly dictated by economic drivers in interaction with factors influencing wood volume growth, such as tree species and site productivity. However, in many parts of the world, forest management needs to consider an increasing range of forest values beyond traditional wood products, such as recreation, biodiversity conservation, and climate change mitigation.

Considering the increasing variety of demands put on forests and the need to adapt forestry to changing conditions (e.g. climate, economy), rotation lengths are unlikely to remain constant in the future. Over recent decades, rotation lengths have been shortened in the coniferous forests of the northwestern USA (Curtis 1997), New Zealand’s radiata pine (*Pinus radiata*) plantations (from 40–50 years in the 1970s to 25–30 years in the 1990s; Brockerhoff et al. 2008), and the beech (*Fagus sylvatica*) forests of Central Europe (Lange et al. 2014), to mention a few examples. In addition to these observed trends, various stakeholders in the forest sector increasingly argue for modifying forest rotation lengths in the future. For example, the largest forest owner association in southern Sweden recently recommended to shorten rotations by 10–15 years in Norway spruce (*Picea abies*)-dominated forest (where typical rotations are ~60–80 years) to decrease the risk of storm and root rot damage (Södra Skog 2012). In some countries, a minimum allowable felling age is specified in the legislation. In Sweden, for instance, this minimum age varies from 45 to 100 years depending on site productivity and geographic location (Fries et al. 2015). However, to ensure wood supply in spite of increasing protection of older forest with high conservation value, the Swedish state forest company has lately expressed a desire to be allowed to harvest some types of forest stands earlier than currently permitted by law (Fries et al. 2015). Moreover, to improve logistics and conditions for the forest industry, the Finnish government recently abolished regulations imposing a minimum felling age (Fries et al. 2015). In parallel, policy tools have also been developed to provide incentives for longer rotations, e.g. the temporary ‘conservation contracts’ in Fennoscandia (Mönkkönen et al. 2009) as well as ‘ageing forest islands’ in continental Europe (Lassauce et al. 2013) and North America (Jetté et al. 2013) used to promote biodiversity conservation. Considering these recent trends, it is crucial to understand how modified rotation lengths may influence the variety of values attributed to forests.

Most past studies about rotation lengths have focused on economic aspects of wood production; very few have addressed the effects on several forest-based values simultaneously (see however Weslien et al. 2009; Zanchi et al. 2014). The present paper aims to review the socio-ecological implications of modifying rotation lengths by evaluating potential effects on a wide range of ecosystem services and on biodiversity conservation (both concepts sensu MEA 2005). We use boreal Fennoscandian forestry as a case providing a common platform for these analyses. Fennoscandia is characterized by a long history of forest management, the majority of productive forest land having been actively managed for wood production for at least a century. This region is particularly suited as a case because of (1) the dominance of even-aged management systems since the 1950s, and (2) a long tradition of research addressing economic, ecological and social aspects of forest management.

We use an interdisciplinary approach to evaluate the potential effects of shortened and extended rotation lengths on provisioning ecosystem services (wood production, berry and mushroom yields, as well as forage for hoofed game and reindeer), regulating services (control of biotic and abiotic damage to forest resources, climate change mitigation), supporting services (hydrology, water quality and soil nutrients), cultural services (aesthetics and recreation, cultural heritage protection) and biodiversity conservation. The latter is considered a foundation for all ecosystem services (MEA 2005). The studied ecosystem services and biodiversity implications capture a breadth of issues that are common to many other forested regions worldwide, making our case internationally relevant. Building on the Fennoscandian case, we then discuss the implications for forest landscape planning and highlight the key knowledge gaps.

## Benchmark rotations and delimitation of the study

To evaluate the implications of modified rotation lengths, there is a need to define a benchmark for comparison. Here, we use the economically optimal rotation length for wood production based on a discounting rate of 2.5 % as a benchmark representing current practice in Fennoscandian forestry (Simonsen et al. 2010). Considering current silvicultural costs and timber pricelists, this generally represents rotation lengths in the range 60–120 years in boreal Fennoscandia, depending on site productivity and tree species. Hereafter, we use the adjective “mature” to depict a stand which has reached an age corresponding to the benchmark rotation length for a given site productivity and tree species.

To evaluate the potential implications of modified rotation lengths, we reviewed the literature about each of the covered ecosystem services (Table 1), as well as about substrates, habitats and landscape properties of importance to biodiversity conservation (hereafter ‘biodiversity indicators’; Table 2). In view of limited knowledge, we evaluated qualitatively the general directions of changes in ecosystem services and biodiversity that can be expected from modified rotation lengths, without attempting to quantify the effects of rotation-length changes of specific magnitudes. Still, for shortened rotation scenarios, we restricted our analysis to rotations which are shorter than what would result solely from adaptation to future increases in tree growth due to tree breeding, forest fertilization or climate change (Bergh et al. 2010). Hence, in terms of within-stand structure, the evaluated shortened rotations are expected to yield trees which have a smaller diameter at final felling than under the benchmark scenario. Extended rotations are expected to yield a larger maximum tree diameter, as well as more time for accumulation of dead wood and undergrowth of shade-tolerant trees and shrubs. A general implementation of shortened rotation means that at a given point in time, a larger proportion of the landscape will belong to the clearcut and young forest phases. Conversely, longer rotations imply smaller proportions of the landscape in the clearcut and young forest phases, as well as the presence of post-mature forest stands in the landscape.

**Table 1:**
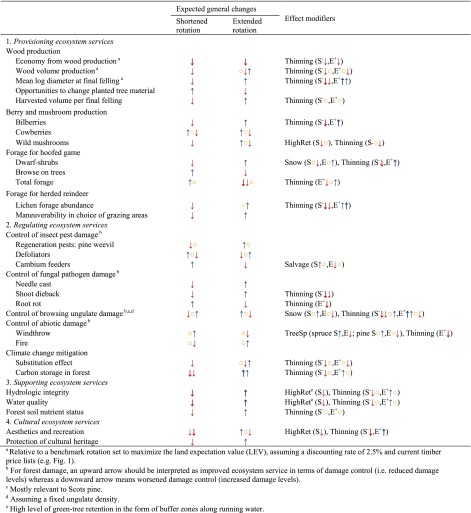
Expected implications of shortened and extended rotation lengths on ecosystem services. Symbols indicate expected general changes relative to the benchmark rotation length: no change (), increase () or decrease () in ecosystem service. Bold arrows (,) indicate a stronger effect than plain arrows. Effect strength is only comparable within a given row and within one arrow direction. Combinations of several symbols depict multiple possible effects depending on the magnitude of rotation-length change or due to uncertainty. The symbols are presented in tentative order of estimated commonness or likelihood. The expected general changes pertain to the average situation over a complete rotation without modification of the thinning regime, and include landscape-scale effects wherever relevant. The rightmost column presents key effect modifiers: (1) potential adaptations of the thinning regime as a response to changed rotation length (‘Thinning’), (2) high levels of tree retention at final felling (‘HighRet’), (3) systematic salvage logging of windthrown trees within stands (‘Salvage’), (4) main tree species forming the stand (‘TreeSp’) and (5) presence of a thick snow cover during winter (‘Snow’). Whenever different from the general patterns, we present the expected effects of shortened (‘S’) or extended (‘E’) rotations under each of these specific conditions. For thinning, ‘S^−^’ depicts a shortened rotation where the stand is subjected to fewer thinnings (or remains unthinned) during its growth as an adaptation to the shorter stand growth period, and ‘E^+^’ depicts an extended rotation where the stand is subjected to additional thinning(s) as an adaptation to the longer stand growth period

**Table 2:**
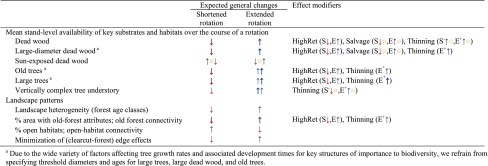
Expected implications of modified rotation lengths on biodiversity conservation. Explanations as in Table 1

Production forests in boreal Fennoscandia are dominated by the two native conifers Scots pine (*Pinus sylvestris*) and Norway spruce. To better isolate the effects of rotation lengths per se, we focused on stands which are dominated by these two native tree species during most of the rotation (although the stands may comprise some proportion of deciduous trees especially at the sapling stage), assuming that changes to rotation lengths are not accompanied by changes in the identities of the species forming the main tree cohort. Hence, we did not evaluate the effects of shortened rotations stemming from the use of faster-growing non-native species or hybrids, neither does our evaluation encompass ‘short-rotation forestry’ systems for energy crops (e.g. willow *Salix* spp. coppice).

## Effect modifiers

The effects of altered rotation lengths on some of the studied forest values were found to be modified by a number of factors, especially thinning. Modifications of rotation lengths *per se* may motivate changes to the thinning regime, with potential impacts on a range of forest values. If rotations are shortened, the economically optimal number of thinnings may be lower than under the benchmark rotation, and vice versa for extended rotations. Our main results (*cf*. the two central columns in Tables 1 and 2) present the main expected effects of modified rotations under unchanged thinning regimes. However, wherever relevant, we report how changes to thinning regimes (implemented as an adaptation to the new rotations) may modify the effects of changed rotations lengths. In addition to thinning, the following factors were found to have a particularly important influence on the effects of changed rotations: high levels of tree retention at final felling, salvage logging of dying and dead trees, main tree species forming the stands, and local climatic conditions (snow cover). Hence, for each of the studied ecosystem services and biodiversity indicators, we report—wherever relevant—how their relation to rotation length is influenced by these effect modifiers (*cf*. rightmost column in Tables 1, 2).

## Implications of modified rotation lengths

### Provisioning ecosystem services

#### Wood production

**Fig. 1:**
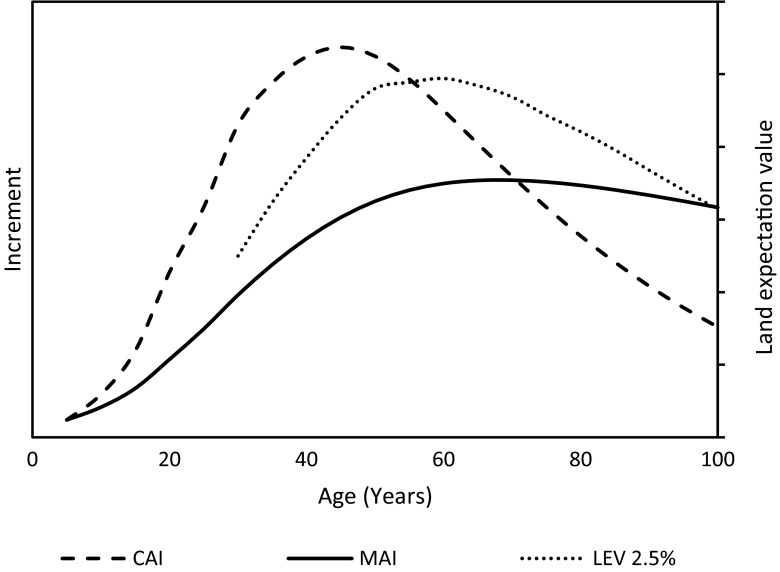
Current annual increment (CAI) of stand volume at different forest stand ages (dashed curve), mean annual increment (MAI) since stand establishment (plain curve), and land expectation value (LEV) for rotations of different lengths, based on a 2.5 % discounting rate (dotted curve). This example is based on the simulation of a Norway spruce stand’s development using the Heureka software (Wikström et al. 2011). The economically optimal rotation length for wood production is ~60 years (culmination of LEV), while wood volume production is maximized at a rotation of ~70 years (culmination of MAI)

Wood volume growth of managed even-aged stands follows a pattern whereby the current annual increment (CAI) rises after stand establishment, peaks when maximum leaf area is attained and then declines (Assmann 1970; Fig. 1). The CAI curve and the resulting mean annual increment (MAI) curve (i.e. average annual increment since stand establishment) have shapes which vary according to tree species and site conditions. The two curves intersect where the MAI culminates. This is the best time for harvesting a stand if the aim is to maximize wood volume production.

However, forest management decisions are not only driven by wood volume considerations, but also by economy and other factors. Faustmann (1849) proposed a method to calculate the economically optimal rotation length through optimization of the land expectation value (LEV). Today, this method is widely accepted. By combining general growth patterns of even-aged stands and the Faustmann method, it can be shown that economically optimal rotations are shorter under higher interest rates. A strong time preference for income in the near future (as opposed to the distant future) and high uncertainty are typically associated with high interest rates. The economically optimal rotations are also shorter if the price premium for large-diameter sawlogs is low.

In managed Fennoscandian forest stands, the LEV curve is typically asymmetrical around the apex (Fig. 1), implying higher economic loss when the rotation is reduced rather than prolonged by a given number of years (not considering potential changes in the risk for catastrophic damage). The negative economic effects of altering rotation length can be moderated to some extent by adjusting the thinning programs to the planned rotation length, i.e. thinning less for shorter rotations and more for longer rotations. Under currently prevailing conditions in Fennoscandia—including our benchmark scenario—LEV usually culminates earlier than MAI (Fig. 1). This means that extending the rotation by a limited amount of time after the benchmark economic optimum will likely maintain (or slightly increase) wood volume production, while a shortening or a substantial extension will result in volume loss (although these effects can be modulated by adaptations of the thinning regimes; Table 1). Note that this assessment does not include potential effects of large-scale damage incurred by e.g. windthrow or insect outbreaks. These are considered in a separate section below.

Longer rotations generally imply a larger harvested volume per final felling and hence potentially more efficient logistics, an economic advantage typically not included in the LEV. At the same time, extending rotations may reduce wood supply to the industry during the period of transition to the new rotation length (Curtis 1997). Moreover, due to longer time between regeneration occasions, longer rotations offer less frequent opportunities for changing management strategies (e.g. choice of planted tree material) in the face of improved silvicultural knowledge or climate change.

#### Berry and mushroom production

In Fennoscandia, forest berries (mainly bilberry *Vaccinium myrtillus* and cowberry *V. vitis*-*idaea*) and edible mushrooms have large economic (Yun and Hall 2004) as well as cultural and recreational values (Kardell 1980). Simulations suggest that compared to producing timber only, optimizing management for the joint production of timber and bilberries would require longer rotations (by up to four decades, depending on bilberry prices) and more frequent thinnings (Miina et al. 2010). These models suggest that shortened rotations could be detrimental to bilberry production and that reducing the number of thinnings as an adaptation to shorter rotations may exacerbate these negative effects. For cowberry, relations to forest age and tree density are less pronounced and somewhat varied (Kardell 1980; Nieppola 1992), making predictions regarding effects of rotation lengths on cowberry production uncertain (Table 1).

Regarding mushrooms, relatively little is known about the effects of forest management on their persistence and productivity. Many edible fungi are ectomycorrhizal and thus depend on the trees’ photosynthesis for their carbohydrate supply. Accordingly, ectomycorrhizal edible mushrooms may largely disappear following clearcutting, to re-establish as the new stand grows (Perry et al. 1987). Shortening rotations across a landscape is thus expected to decrease the availability of edible ectomycorrhizal mushrooms due to more frequent clearcutting, although the effect might be modulated if many trees are retained at harvesting. It has been speculated that mushroom productivity is promoted in dense forest stands because an open tree canopy results in a too dry forest-floor microhabitat (Pilz et al. 2006). Therefore, decreasing the number of thinnings as an adaptation to shortened rotations might alleviate some of the expected negative impacts of more frequent clearcutting. Mushroom productivity in coniferous forest stands in the Pyrenees has been shown to increase over time during stand development, peaking at approximately the same time as the CAI (Bonet et al. 2010). Hence, the effects of extended rotations are unclear because they will depend on the net result of two counteracting factors whose implications are poorly known: an enhanced persistence of ectomycorrhizal fungi (due to less frequent clearcutting) and an increased proportion of stands which are overmature from a mushroom production perspective.

#### Forage for hoofed game

Ungulates are the most important game species in Fennoscandia. For deer, tree twigs, buds and bark are a main food source during winter, dwarf-shrubs such as bilberry and heather (*Calluna vulgaris*) form an important food resource year round (e.g. Mysterud et al. 2010; Tinoco Torres et al. 2011) and leaves from young trees, forbs and grasses are important during summer. The available forage is largely concentrated in space and time in regenerating young stands. The amount of forage for moose (*Alces alces*) can be four times as large during the earlier phases of the rotation compared to later phases (Wam et al. 2010).

Clearcutting and subsequent soil scarification favour grasses and natural recruitment of deciduous trees, but disfavour dwarf-shrubs such as bilberry (Hedwall et al. 2013). Extending rotations would give dwarf-shrubs more time to recuperate after clearcutting, and hence provide ungulates with increased access to forage in older stands, especially if the stands are well-thinned and snow cover does not restrict access to the dwarf-shrubs in winter. However, extended rotations would decrease the proportion of younger forest and hence the landscape-scale availability of browse trees compared to shorter rotations. As young stands hold more forage than older stands (Wam et al. 2010), the overall availability of forage would probably be higher under shortened rotations (Table 1).

#### Forage for herded reindeer

Reindeer (*Rangifer t. tarandus*) herding is practiced over most of northern Fennoscandia. During the winter, semi-domesticated reindeer graze mostly in forest (Kivinen et al. 2010), where ground lichens (mainly *Cladonia* spp.) and arboreal lichens (mainly *Alectoria* and *Bryoria* spp., which are most abundant on old trees) constitute the main food source. Shortened rotations are expected to have predominantly negative impacts on reindeer husbandry (Table 1). First, they involve more frequent clearfelling, which causes a direct loss of arboreal lichens, and reduces the time window for recolonization by these lichens (Dettki and Esseen 2003). Shortened rotations also imply more frequent soil scarification, with negative impacts on ground lichens (Roturier and Bergsten 2006). Kivinen et al. (2010) recommend more intensive thinning as a way to favour lichens and facilitate herding of the reindeer. Hence, decreasing the number of thinnings as an adaptation to shortened rotations would likely exacerbate these negative impacts.

Conversely, extended rotations may benefit reindeer forage by reducing lichen loss, allowing more time for lichen recolonization, and reducing the landscape proportion of dense young forest with poor ground lichen growth (Kivinen et al. 2010). However, to yield substantial positive effects, the extension would probably need to be very long and ideally accompanied by additional thinning. Longer rotations also increase the diversity of forest stand age classes in the landscape, which gives reindeer herders more flexibility in finding alternative grazing areas when weather-related changes in snow conditions affect access to ground lichens (Horstkotte et al. 2014).

### Regulating ecosystem services

#### Control of insect pest damage to forest

Insect pests with especially large impacts on Fennoscandian forestry include regeneration pests, defoliators and cambium feeders. The most damaging regeneration pest is the pine weevil (*Hylobius abietis*), affecting both pine and spruce seedlings. Shorter rotations imply that a given site is more often in the regeneration stage, which may increase the importance of pine weevil damage, since the weevil depends on the availability of stump roots after final felling (Björkman et al. 2015). Extended rotations are expected to have an opposite effect.

Regarding defoliating insects, some empirical studies (e.g. De Somviele et al. 2004) have found that young trees are less damaged, possibly because of higher concentrations of defensive compounds compared to older trees (Bryant et al. 1991). However, the effects of rotation lengths on defoliator damage are difficult to generalize because (1) different defoliating species seem to respond individually (Björkman et al. 2015) and (2) other factors, such as stand characteristics and site conditions, seem to affect density and damage more than stand age (Charbonneau et al. 2012).

Among cambium feeders, the most damaging insects in Fennoscandia are the spruce bark beetle (*Ips typographus*) and—to a lesser extent—the pine shoot beetle (*Tomicus piniperda*). Shortened rotations would most likely decrease, and extended rotations increase, damage by these species because the amount of suitable breeding material is expected to increase with tree age (Eidmann 1992; Björkman et al. 2015). However, these effects may be altered by salvage logging of windthrown or otherwise dying trees within stands (Table 1).

#### Control of pathogen damage to forest

Forest fungal pathogens affect stands at all development stages, but some are specific to a certain tree age class. Under shorter rotation scenarios, diseases affecting young trees will probably increase in importance while those causing damage in old forests will decrease (Table 1). Hence, needle cast disease (e.g. *Lophodermium seditiosum*) and shoot dieback (e.g. *Gremmeniella abietina*, which periodically causes major damage especially to pine in Fennoscandia) can be expected to increase in importance under shortened rotations because they affect mostly young to middle-aged forest (Capretti et al. 2013). Root rots are the economically most important fungal diseases in Fennoscandia (Oliva et al. 2010). Damage is likely to increase with extended rotation time because root rot, which spreads gradually in the wood of infected trees as they age, will have more time to reach and affect the stem.

Changes to the thinning regime as an adaptation to changed rotations may alter the magnitude of the effects of rotation length on pathogen damage. Spruce root rot caused by *Heterobasidion* spp. is highly dependent on thinning stumps for its dynamics (Thor and Stenlid 2005) and hence damage may increase if prolonged rotations involve additional thinnings. In contrast, thinning may help control several shoot- and leaf-infecting fungi which are favoured by dense stands with high humidity and short dispersal distances between trees. For example, *G. abietina* damage can be partly prevented by thinning pine stands (Niemelä et al. 1992). Refraining from thinning when shortening rotations may increase that type of damage.

#### Control of ungulate browsing damage to forest

For a given ungulate density, browsing damage to commercially valuable trees generally decreases with increasing forage availability (Månsson 2009). However, the type of forage available in the landscape also plays a role. Damage from moose on young Scots pine occurs mostly in the winter and is less severe in years with little snow cover (e.g. Månsson 2009), which can be attributed to moose feeding more on bilberry in older stands. Shortened rotations are expected to reduce bilberry cover (see above) and may thereby concentrate forage and thus hoofed game to the young stands, where trees are susceptible to browsing damage. This may increase forest damage, even if the total amount of forage increases due to larger areas of young stands. Hence, the net effect of rotation-length modifications is unclear and may depend not only on how they influence the area of forage-rich young forest, but also on how site conditions, silvicultural practices and weather (snow cover) affect the availability of alternative sources of forage in older stands (Table 1).

#### Control of abiotic forest damage

Future climate change is expected to increase the vulnerability of Fennoscandian forests—especially spruce-dominated stands—to windthrow (Blennow et al. 2010). In even-aged forest, storm damage generally increases with stand height and age, and recent thinning is an additional risk factor (Mitchell 2013). Hence, extending rotations would likely increase wind damage, especially if the extension implies additional thinning at a later stage. In contrast, shortening rotations may help limit such damage (Valinger and Fridman 2011).

Fire is another major abiotic disturbance in boreal forests. The risk for fire damage depends on climate, fuels and ignitions, of which the latter two are influenced by land management. Any alteration to cutting regimes will directly impact the structure of both the surface and canopy fuel layers, thereby altering fire risk. In the first few years after final felling, the surface fuels are abundant because of the addition of logging slash that follows the typical cut-to-length felling system employed in Fennoscandia. They are then also highly flammable since they are fully exposed. Shortened rotations will therefore increase (and extended rotations decrease) the proportion of the landscape that is in a highly flammable condition. Also, forestry machinery is in itself a relatively frequent fire starter, especially in stony areas. Once stand closure is completed, further changes in flammability with age may be relatively small, but have not been assessed for Fennoscandia, except for post-fire succession (Schimmel and Granström 1997). Changes to the thinning regime as an adaptation to altered rotations may also possibly influence fire risk, but the many factors involved (development of understory vegetation, fuel drying, probability of transition from surface to crown fire; *cf*. Agee and Skinner 2005; Wotton and Beverly 2007) preclude any clear predictions. All things considered, the net effect of modifying rotation lengths on fire risk at the landscape scale is likely to be relatively modest and uncertain (Table 1).

#### Climate change mitigation

Tree growth provides an efficient means of removing CO_2_ from the atmosphere and producing raw materials to substitute fossil fuels. Moreover, forests constitute large carbon stores: increased tree biomass and carbon stored in the soil also means less CO_2_ in the atmosphere (Poudel et al. 2011). Thus, the forest sector contributes to climate change mitigation in two ways: fossil fuel substitution and carbon storage. An important difference is that the displacement of fossil fuels results in a permanent benefit while the carbon storage option is temporary (Lundmark et al. 2014). A number of studies suggest that longer rotations increase the average carbon stock in stands (e.g. Kaipainen et al. 2004; Zanchi et al. 2014). Although the effects of rotation length are clear regarding carbon stock in the standing biomass, ambiguous results about the effects of forest management on soil carbon preclude firm conclusions for soil carbon stocks (Clarke et al. 2015). In a managed forest landscape, the likely positive effects of extending rotations on carbon storage might be countered in the short term by reduced harvesting during the period when the carbon stock is increased in the forest (Curtis 1997), resulting in temporarily lowered substitution effects. Shortened rotations would likely decrease average carbon stock in stands, although increased harvesting in the landscape during the transition period may temporarily benefit substitution. The overall long-term effects of changed rotation lengths on substitution are tightly linked to how these alterations affect average wood volume production (*cf*. “Wood production” above). If final felling takes place before the culmination of the MAI (as is the case for rotations shorter than under our benchmark scenario), the average wood volume yield and hence the substitution effect will be reduced, with negative impact on climate mitigation. Changes to thinning regimes allowing higher stocking would be positive for carbon storage and may also increase the wood volume yield compared to more intensive thinning (Garcia-Gonzalo et al. 2007; Table 1).

### Supporting ecosystem services

#### Hydrology, water quality and soil nutrients

Although changes to rotation lengths may have both hydrological and biogeochemical consequences, the effect will primarily be a reinforcement (shortened rotations) or reduction (extended rotations) of those associated with conventional forest management (Table 1). From a soil and stream water perspective, the main forestry impacts arise from (1) a physical interruption of the hydrological cycle, and (2) more direct biogeochemical effects in the soil. The removal of the tree canopy leads to a decline in ecosystem transpiration, which results in wetter soils and increased stream runoff (Andreassian 2004). The hydrological impact is largest in the years following harvesting (or thinning) and decreases gradually with tree canopy recovery (Hubbart et al. 2007). Shortened rotations involve more frequent harvesting and should therefore increase negative hydrological impacts, while the opposite can be expected for extended rotations. While wetter soils result in increased risk of rutting caused by heavy machinery, higher runoff results in elevated concentration and larger stream export of dissolved organic carbon (DOC; Schelker et al. 2012), mercury (Bishop et al. 2009), and dissolved inorganic nitrogen (DIN; Kreutzweiser et al. 2008). The role of DOC is particularly important in boreal landscapes, not only because DOC represents an important flux component to the regional carbon cycle (Öquist et al. 2014), but also because DOC acts as a primary transport-vector for contaminants such as mercury (Bishop et al. 2009). Transformation of total mercury to its toxic methyl mercury form is enhanced in water-logged soils (Skyllberg et al. 2009). The expected negative impacts of shortened rotations on hydrologic integrity and water quality could be mitigated to some extent by the retention of more extensive buffer zones along streams.

Tree harvesting will result in a reduction of the base cation pool in the soil (Akselsson et al. 2007), and modelling results by Zanchi et al. (2014) suggest slightly decreased acid neutralizing capacity as a result of shortened rotations. Whether that will affect the buffering capacity of streams remains unclear (Futter et al. 2012), but if there is such an effect a shortening of the rotation period could have serious implications on pH in weakly buffered streams (Ågren et al. 2010). While a change in the length of rotation periods in the future would not necessarily cause additional stream water quality impairments on the local scale, it may have downstream consequences (Laudon et al. 2011). As river systems are unidirectionally connected, any upstream impact will result in downstream perturbation unless the effect is diluted or the solute in questions is remobilized by instream processes. While relatively little is known about those effects, shorter rotations imply that more of the catchment is harvested every year, potentially resulting in larger downstream perturbations.

### Cultural ecosystem services

#### Aesthetics, recreation and cultural heritage

Forest management directly affects aesthetical and recreational values. Although preferences vary among people and cultures (e.g. Lisberg Jensen and Ouis 2014), a forest stand’s attractiveness for recreational activities generally increases with the size of the trees, and consequently old forests are usually preferred to young stands in Fennoscandia (e.g. Gundersen and Frivold 2008). Furthermore, preference studies show that the absence of clearcuts and other obvious traces from forest management is important to forest visitors (e.g. Gundersen and Frivold 2008; Eriksson et al. 2012). Studies of Swedish spruce forests suggest that the part of the rotation having the lowest recreational value is when the stand reaches a height above the eye level and enters the dense sapling stage (Gustafsson et al. 2009). Shortened rotations are expected to impact aesthetic and recreational values negatively due to larger landscape-scale proportions of clearcuts and dense young stands at the expense of old forest, while extended rotations should have positive effects (Curtis 1997) (Table 1). However, high levels of green-tree retention may alleviate to some extent the negative aesthetic impacts of more frequent clearcutting under shortened rotations (Ribe 2005).

In Fennoscandian preference studies, thinned and cleaned forests with semi-good visibility score higher than forests with a dense understory and forests with dead wood on the ground (e.g. Holgén et al. 2000; Eriksson et al. 2012). The recreational value rises after a first commercial thinning, especially if slash is removed from the site (Gustafsson et al. 2009). Hence, reducing the number of thinnings as an adaptation to shortened rotations is expected to exacerbate negative impacts on aesthetics and recreation. Moreover, due to dead wood accumulation and undergrowth of shade-tolerant trees, refraining from performing additional thinning in stands subjected to extended rotations (e.g. when temporary setting aside mature or overmature stands for biodiversity conservation) may counteract to some extent the positive effects of increasing the amounts of older forest in the landscape. Therefore, the overall effect of extending rotations on aesthetics and recreation remains uncertain and largely dependent on the specifics of dead wood accumulation and undergrowth (Table 1).

Alterations to rotation lengths may also affect cultural heritage. In Fennoscandia, ancient monuments and historic sites in forest (e.g. remnants of ancient settlements, graves and sawmills) are protected by law. Yet, a significant number of remnants are damaged or destroyed during forestry operations. Due to more frequent soil scarification and machine driving, shorter rotations are expected to increase the risk of physical damage to such objects (Fries et al. 2015), while longer rotations may help reduce this risk.

### Biodiversity conservation

In the absence of human intervention, Fennoscandian boreal forests would be shaped by a mixture of large-scale disturbance-succession processes (following e.g. fires) and processes operating at a smaller grain (e.g. gap dynamics; Angelstam and Kuuluvainen 2004). As a result, highly heterogeneous forest environments historically characterized the forest landscapes, which included the regular occurrence of old trees and the presence of areas where forest cover was maintained more or less continuously over time (Angelstam and Kuuluvainen 2004). Systematically shortening rotations throughout the landscape is inconsistent with aims to better emulate natural disturbance regimes, as it curtails forest stand development at an age younger than would be found in substantial parts of naturally dynamic forest landscapes. In contrast, extending rotations on some proportion of the land base can be expected to alleviate some of the differences between managed and naturally dynamic forest landscapes (Burton et al. 1999; Lindenmayer and Franklin 2002).

Old forest supports microhabitats that are rare or absent in younger forest (e.g. Lassauce et al. 2013) and critical to large numbers of red-listed forest species (Bernes 2011). Important microhabitats on old or large trees include cavities, thick creviced bark, exposed wood, fungal fruiting bodies and mycelium, large branches and dead roots (Siitonen 2012). In older stands, dead wood accumulates more quickly as a result of increased tree mortality and the diameter of the dead wood is larger (Jonsson et al. 2006). Therefore, landscape-scale habitat availability for species specialized on old-tree microhabitats and larger-diameter dead wood may increase if rotations are extended (Dettki and Esseen 2003; Jonsson et al. 2006). However, some structures (e.g. very large tree hollows created by wood decay) develop at such a late age that they do not occur in trees grown for timber production (Lindenmayer and Franklin 2002). Shorter rotations imply more frequent clearfelling and soil scarification events, which typically result in the destruction of a large proportion of the dead wood pool (Hautala et al. 2004). In addition, shorter rotations allow less time for the gradual development of an understory of shade-tolerant trees (e.g. Hansen et al. 1995), with possible negative impacts on species dependent on a vertically complex vegetation structure. However, potential changes to thinning regimes associated with rotation-length modifications also need to be considered. Indeed, thinning may influence habitat quality via reduced tree density and mortality (and hence reduced dead wood accumulation), increased growth of residual trees, reduced vertical heterogeneity of the main tree layer, changes to understory structure, and altered micro-climatic conditions. Moreover, salvage logging of trees damaged by wind, insects or pathogens within stands may reduce some of the conservation benefits of extended rotations by preventing dead wood accumulation (Żmihorski 2010). Generally, extending rotations without additional thinning or salvage logging seems to be the most beneficial rotation scenario for species dependent on old-forest attributes such as dead wood (Table 2).

Some of the negative impacts of shortened rotations on biodiversity can be partly alleviated through specific conservation measures, such as retention of green trees and dead wood at clearfelling (Lindenmayer and Franklin 2002). The absolute contribution of tree retention to the provision of large and old trees may be larger under shorter rotations, because more frequent opportunities are provided to replace blowndown retention trees with new ones (Nilsson et al. unpubl.).

The different stand-scale effects of altered rotations translate to the landscape scale. At a given point in time, the proportion of forest containing older-forest attributes will decrease if rotation lengths are systematically shortened, thereby potentially reducing the connectivity of such habitat. Moreover, in each stand subjected to shortened rotations, the suitable temporal window for old-forest associated species becomes narrower, which decreases the time available for colonization, reproduction and dispersal (Dettki and Esseen 2003). Reduced spatial connectivity and shorter temporal persistence of old-forest habitats may increase the extinction risk of old-forest specialists. In addition, shortening rotations results in increased amounts of edges between open habitat and forest, with potentially detrimental effects on edge-sensitive species (Harris 1984).

Shortened rotations may, however, improve the situation for some species by increasing the availability of open habitat (Hansen et al. 1995). Provided that sufficient amounts of dead wood are retained at harvesting, threatened beetle (Coleoptera) species dependent on sun-exposed wood could benefit from increases in the amounts of open areas and edges (Rubene et al. 2014). Whether shortened rotations would increase, retain or decrease the amount of sun-exposed dead wood is however unclear, because the availability of that substrate is the product of the expanse of open areas and the amount of dead wood, two factors which may be influenced in opposite directions by a given change in rotation length (Table 2). Acknowledging potential positive effects of shortened rotations on open-habitat species, the provision of old-forest structures and habitats is generally considered a higher conservation priority in today’s Fennoscandian landscapes (Bernes 2011). Hence, extending rotations is expected to be more widely consistent with current biodiversity conservation objectives in Fennoscandia, at least if the extension is substantial enough to allow the development of old-forest attributes.

## Discussion

### Implications of modifying rotation lengths in forest landscapes: Synthesis

Our analyses showed a wide range of effects of modifying rotation lengths on different ecosystem services and on biodiversity conservation. For wood production, both a shortening and extension of rotations would lower economic outputs compared to current practice (assuming no extensive forest damage), although the two strategies may yield diverging effects on harvesting logistics and the provision of specific wood products. The effects of shortening rotations on other provisioning services are expected to be negative to neutral in most cases (e.g. bilberries, mushrooms, reindeer forage), while the effects of extending rotations on these services would be mainly positive or uncertain.

For regulating services, it is difficult to draw any general picture. Shortening rotations may help limit damage by some of today’s most important damaging agents (e.g. root rot, cambium-feeding insects, windthrow), but may also increase other types of damages (e.g. pathogens affecting mostly young trees, regeneration pests). As regards climate change mitigation, significantly shortening rotations would likely be detrimental to both carbon storage and fossil fuel substitution. Substitution is deemed most important as its benefits exceed those of carbon stock changes in the long term (Lundmark et al. 2014). Hence, the net effects of modifying rotations on climate mitigation would be largely dependent on how it would influence the average wood volume yield.

For the three supporting services and the two groups of cultural services evaluated, the pattern was largely consistent: in nearly all cases they were influenced negatively by shortened rotations and positively by extended rotations. As regards biodiversity conservation, most of the forest structures and habitats of importance to threatened species would likely be influenced negatively by shortened rotations and positively by extended rotations, although species linked to open habitats and edges would respond conversely. These general patterns are consistent with the results of a simulation study by Koskela et al. (2007), which showed that including society’s requirements for biodiversity conservation increases the optimal rotation length compared to the Faustmann case.

Importantly, we also found clear indications that the effects of changed rotation lengths on several of the ecosystem services were dependent on the specific conditions under which they were implemented. Especially, changes to thinning regimes introduced as an adaptation to altered rotation lengths often moderated or enhanced the effects of rotation-length changes. Hence, an ad hoc shortening of the rotation in a stand hitherto conventionally thinned will not necessarily yield the same effects as a planned shortening of rotation with reduced or no thinning. Also, extending rotations under a free development philosophy (e.g. temporary set-asides) may have different effects compared to planned continuous management for wood production involving additional thinning(s). In addition to thinning, several other effect modifiers were identified as particularly important: tree retention at harvesting, salvage logging, tree species and snow cover. Although the expected modifying effects of some of these factors were clear, our analysis also suggested that there are significant gaps in knowledge as to how these and other ecological and silvicultural factors may modulate the effects of changed rotation lengths on ecosystem services. For example, the effects of changed rotations on the capacity to control ungulate browsing damage are subject to much uncertainty due to complex relationships between forage availability and the distribution of the ungulates in the landscape, both of which, in turn, depend on specific ecological settings and silviculture (Månsson 2009).

All things considered, our evaluation based on Fennoscandian conditions illustrates that the expected benefits of shortening or extending rotations for specific ecosystem services will necessarily be accompanied by varied and often contrasting effects on other services. This stresses the need for improved communication and structured dialogue among stakeholders to develop and implement landscape-scale rotation-length strategies which maximize possible synergies and minimize conflicts related to the provision of different ecosystem services.

Several authors have proposed implementing a variety of rotation lengths within forest landscapes (e.g. Burton et al. 1999; Seymour and Hunter 1999; Lindenmayer and Franklin 2002). To better emulate natural disturbance regimes, Burton et al. (1999) and Seymour and Hunter (1999) suggested management strategies aiming for tapered forest age-class distributions, where decreasing proportions of the landscape are allowed to persist to successively older ages, well beyond the maximum age of the ‘classic normal forest’ with a single rotation length. This may involve shortening rotations in part of the landscape while extending them elsewhere. Such differentiation of rotation lengths may facilitate the achievement of multiple societal goals at a larger scale.

The expected positive effects of extended rotation lengths need to be contrasted with those of alternative measures promoting old-forest characteristics, such as the establishment of permanent set-asides. A recent simulation study linking landscape management and species requirements showed that, for several forest species, the optimal combination of forest management measures for a given conservation budget included both extended rotations and set-asides among the range of possible measures (Mönkkönen et al. 2014). From a biodiversity conservation perspective, extending rotations results in networks of ‘floating’ (i.e. temporary) set-asides, which may pose a challenge for species with poor dispersal ability. This stresses the need to plan spatially for old-forest connectivity when implementing extended rotations (e.g. Harris 1984). Also, shortened rotations may prove beneficial to biodiversity conservation in some contexts: allowing final felling in intensively managed younger stands may be preferable to harvesting the equivalent volume in older forest of higher conservation value (Fries et al. 2015).

### The future

Two key challenges for future research will be (1) to gain quantitative knowledge about the effects of rotation modifications of given magnitudes, and (2) to refine knowledge about the effects of changed rotations under the range of ecological and silvicultural conditions characterizing current and future forests. Our evaluation allowed identifying the general directions of changes to forest values that may be expected from shortening or extending rotations. However, it did not provide answers regarding, for example, the quantitative effects of extending rotations by one decade vs. half a century. This is currently hampered by a paucity of empirical studies directly evaluating the effect of specific rotation lengths on biodiversity or ecosystem services other than wood production. Such studies would require large-scale experimental systems including forest stands which belong to different age classes (including post-mature stages) and share a similar origin. In the case of biodiversity conservation, most past studies have compared biodiversity in intensively managed younger forest originating from clearcutting with near-natural, post-mature forest stands that have never been clearcut (see however Martikainen et al. 2000; Nagaike and Hayashi 2004). This knowledge may not be directly applicable to extending rotations in future managed forests. As more intensively managed forest stands reach and pass maturity, we see increasing opportunities for well-planned, large-scale field studies addressing the effects of rotation extension on a range of ecosystem services in actively managed forest. These studies should be designed to cover a variety of ecological conditions (e.g. tree species, site productivity, climate) and silvicultural methods (e.g. thinning regimes, slash and dead wood management, tree retention) potentially influencing the effects of rotation lengths. The resulting improvements in empirical knowledge will have direct value for management, and will also provide input for simulation studies which will be necessary to project the long-term, future effects of rotation-length modification on a range of forest values across entire landscapes.
